# The Emerging Role of RNA Modifications in DNA Double-Strand Break Repair

**DOI:** 10.3389/fmolb.2021.664872

**Published:** 2021-04-29

**Authors:** Sonia Jimeno, Fernando R. Balestra, Pablo Huertas

**Affiliations:** ^1^Departamento de Genética, Universidad de Sevilla, Seville, Spain; ^2^Centro Andaluz de Biología Molecular y Medicina Regenerativa-CABIMER, Universidad de Sevilla-CSIC-Universidad Pablo de Olavide, Seville, Spain

**Keywords:** RNA modification, DNA repair, homologous recombination, DNA resection, R-loop

## Abstract

The correct repair of DNA double-strand breaks is essential for maintaining the stability of the genome, thus ensuring the survival and fitness of any living organism. Indeed, the repair of these lesions is a complicated affair, in which several pathways compete for the DNA ends in a complex balance. Thus, the fine-tuning of the DNA double-strand break repair pathway choice relies on the different regulatory layers that respond to environmental cues. Among those different tiers of regulation, RNA modifications have just emerged as a promising field.

## Double-Strand Breaks Repair Pathways

**FIGURE 1:**
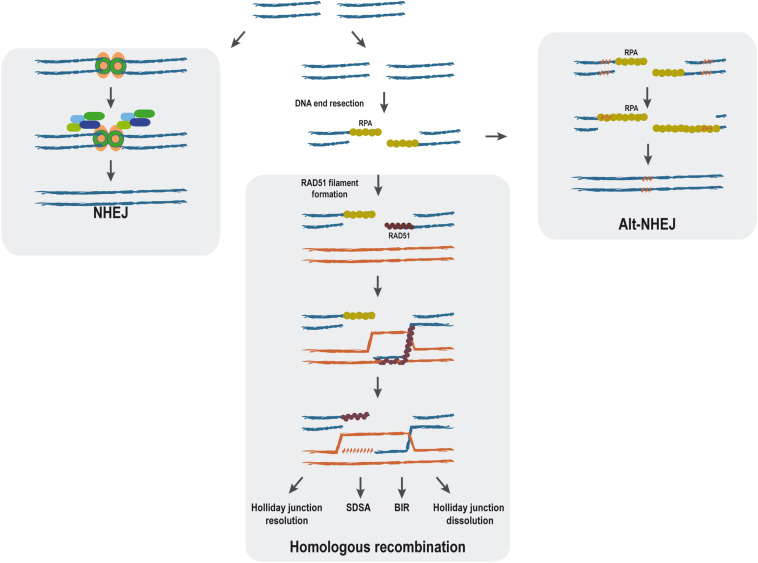
Main double-strand break (DSB) repair pathways. The main steps of non-homologous end joining (NHEJ), alternative non-homologous end-joining, and homologous recombination repair mechanisms are represented. DNA double-strand breaks can be repaired without end processing by NHEJ (left). In response to specific cues, the ends can be processed by a nucleolytic degradation of one strand to form long tails of ssDNA in the process known as resection. When this happens, classical NHEJ is effectively blocked. Sometimes, ssDNA formation will expose short stretches of microhomology (right, punctuated orange pattern), and the break will be repaired by Alt-NHEJ (right). Alternatively, homologous recombination can proceed using the resected DNA. Although HR can proceed through different HR pathways [holiday junction resolution, synthesis-dependent strand annealing (SDSA), break-induced replication (BIR), and holiday junction dissolution], all of them share the initial steps.

When DNA is damaged either physically or chemically, the integrity of the genomic information might be compromised. Therefore, living organisms have many repair pathways that try reverting the damaged DNA to the original DNA sequence or, at least, to minimize the impact of such changes. Particularly difficult-to-manage DNA lesions are DNA double-strand breaks (DSBs), which are produced when both DNA strands break at the same time in close proximity. Such DNA alteration triggers a very complex response, commonly known as the DNA damage response (DDR), that not only initiates the repair of the lesion but causes a complete upheaval of the cellular metabolism (Jackson and Bartek, 2009). In the case of DSBs, several repair pathways coexist. They are classified regarding the use or non-use of extensive homologous sequences as templates (Figure 1). Thus, non-homologous end-joining (NHEJ) requires no homology and process through the religation of both sides of the break (Davis and Chen, 2013; Radhakrishnan et al., 2014), whereas homologous recombination (HR) needs the presence of extensive homologies for the repair (Jasin and Rothstein, 2013). Additionally, alternative non-homologous end-joining (Alt-NHEJ) uses micro-homologous regions. Given that the outcome of each repair pathway is different, the choice between them has to be tightly regulated as errors in this balance can be very deleterious (for a review, see Ceccaldi et al., 2016). It is still not completely understood how cells chose between these alternative pathways, but one of the most important points is the processing of the DNA ends by nucleases to create single-stranded DNA (ssDNA), the so-called DNA end resection step (Figure 1). It consists of the nucleolytic production of long tails of ssDNA, which are promptly covered by the RPA complex that protects the structure (Huertas, 2010; Symington et al., 2014; Cejka, 2015). Such ssDNA is required for HR and Alt-NHEJ but efficiently blocks NHEJ. During classical HR, RAD51 replaces RPA, thus forming the so-called nucleoprotein filament that has the ability to search for homologous DNA (Figure 1) (Daley et al., 2014; Greene, 2016).

Many different aspects of cellular biology impact the outcome of the DSB repair. Among them, it has become clear in recent years that the RNA itself and RNA-related proteins play some essential roles (for review, see Jimeno et al., 2019). In this review, we focus specifically on the emerging significance of RNA modifications for DSB repair.

## Roles of Different RNA Molecules in HR

There is extensive literature on the regulatory effects of different RNAs in DNA repair through *in trans* effects, that is, by affecting the expression of repair factors. Several comprehensive reviews can be found in the literature (Thapar, 2018; Jimeno et al., 2019; Bader et al., 2020). But additionally, RNA can affect directly the DSB repair *in cis* by the action of different RNA species, including the role of specific non-coding RNAs (ncRNAs) or by the presence of DNA:RNA hybrids at the site of DNA breaks.

### Roles of RNA in the Damage-Induced Recruitment of Repair Factors

The first piece of evidence in this regard was shown by Pryde et al. (2005), when they showed that RNase A treatment caused the disappearance of ionizing radiation (IR)-induced 53BP1 foci and, moreover, that this effect was reverted by the addition of ectopic RNA (Pryde et al., 2005). Thus, it seems that RNA might stabilize some IR-induced foci (IRIF). Indeed, DICER and DROSHA play an RNA-mediated role in the secondary recruitment of DDR factors such as MDC1 and 53BP1 and, thus, in the amplification of the DDR signal (Francia et al., 2016). Recent work has allowed di Fagagna’s lab to propose a model in which the assembly of complete promoters at the sites of DSBs drives RNA synthesis, stimulating the phase separation of DDR factors in the shape of foci that exhibit liquid–liquid phase-separation condensate properties (Pessina et al., 2019). However, this does not apply generally to all IRIF, since RNA is not necessary for the formation of foci of the DDR sensor NBS1 (Francia et al., 2016), and, furthermore, the accumulation of RNA has been shown to be detrimental for the formation of RPA foci (Domingo-Prim et al., 2019).

### *De Novo* Transcription of Long Non-coding RNA at DSB Sites

Although it has been thoroughly described that transcription is generally repressed after the appearance of DSBs (Shanbhag et al., 2010; Pankotai et al., 2012; Kim et al., 2015; Manfrini et al., 2015), some labs have reported *de novo* transcription around the DSB (Francia et al., 2012; Ohle et al., 2016; Michelini et al., 2017; D’Alessandro et al., 2018; Burger et al., 2019; Pessina et al., 2019; Vítor et al., 2019; Sharma et al., 2021). Thus, after DNA damage, RNA polymerase II is supposed to be recruited to the damaged chromatin and to start transcription around the break. Such transcription creates *de novo* long lncRNAs. The production of these damage-induced non-coding RNAs (dincRNAs) due to *de novo* transcription at the sites of the breaks is supposed to be essential for the proper activation of the DDR (Francia et al., 2012) and to fulfill the repair of the DSB, mainly by affecting Rad51 recruitment and thus having an impact on HR efficiency (Gao et al., 2014). In agreement, the inhibition of transcription before the damage induction negatively affects the formation of RPA and Rad51 foci (Yasuhara et al., 2018). However, whether the production of these damage-induced ncRNAs depends on paused polymerases rather than *de novo*–loaded ones is still open for debate (Puget et al., 2019).

### Messenger RNA as a Repair Template

Another attractive possibility is that the mRNA molecule might act as a template, that is, a source of homology for the HR repair, but an unequivocal proof of such a role still remains evasive. In budding yeast RNaseH1 and RNAseH2 double mutants, HR can proceed directly using RNA templates, albeit with very low efficiency (Keskin et al., 2014). Indeed, *in vitro*, both yeast and human Rad52 could catalyze direct annealing of RNA to a DSB-like DNA end (Keskin et al., 2014). Furthermore, human and yeast Rad52 and Rad51 show the inverse strand exchange activity (Wahba et al., 2013; Mazina et al., 2017). This DNA–RNA strand exchange activity has also been described for PALB2, which supports not only the forward but also the inverse strand exchange in coordination or in the absence of RAD51 (Mazina et al., 2017; Deveryshetty et al., 2019). This homology-directed RNA–templated DNA repair seems to require transcription to provide the homologous transcript, which in turn forms an active ribonucleoprotein complex with RAD52 (McDevitt et al., 2018). Also, a Cockayne Syndrome group B (CSB)-mediated *de novo* transcription–dependent HR pathway has been found to rely on the capacity of RAD52 to bring an RNA template to the break site (Wei et al., 2015). Moreover, in postmitotic neurons, RAD52 is recruited to DSBs in a fashion that is dependent on the presence of nascent mRNA in the form of an R-loop (Welty et al., 2018). However, not only HR can use the RNA as an intermediate for repair, but even during NHEJ, an RNA molecule could be used to promote error-free repair at transcribed regions (Chakraborty et al., 2016). Furthermore, three additional DNA repair pathways that require the RNA molecule have been described recently in yeast. RNA- and cDNA-templated DSB repair pathways (R-TDR and c-TDR) use either an RNA transcript or a DNA copy of the RNA transcript for repair of induced DSBs, whereas RNA-templated DNA modification (R-TDM) acts on spontaneous or mutagen-induced breaks (Meers et al., 2020). Both R-TDR and R-TDM require the action of the RNA polymerase zeta. Despite the relevance of these findings, it is important to note that both R-TDM and R-TDR are observed only when RNase H1 and H2 are mutated, raising the possibility that these might not be the relevant repair routes in a physiological background.

### DNA:RNA Hybrids on DSB Repair

**FIGURE 2:**
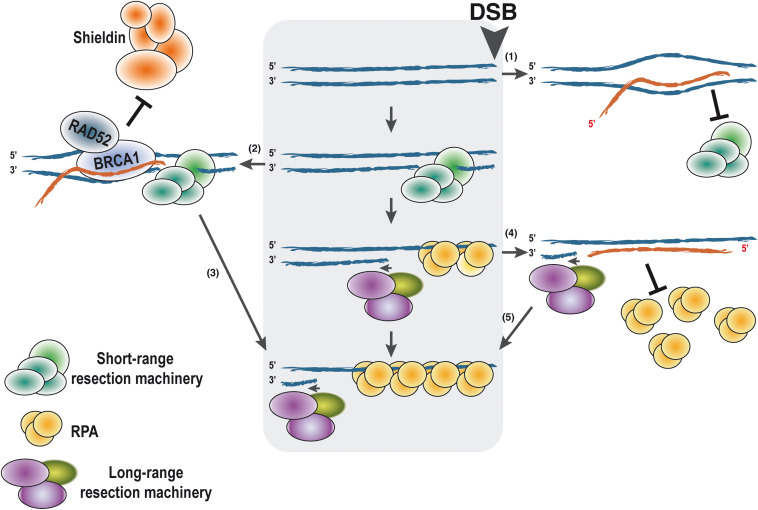
Putative effects of DNA:RNA hybrid presence on DNA end resection according to the timing of their appearance. DNA end resection proceeds unimpaired in the absence of the DNA:RNA hybrid (central gray box). For simplicity, only one end of the DNA is shown, with the DSB on the right side as marked. When an R-loop is present at the site of the break, it might block DNA end resection by blocking the first steps of the process **(1)**. On the contrary, other studies indicate that if resection can indeed be started, the presence of a DNA:RNA hybrid can stimulate resection by attracting RAD52 and BRCA1 **(2)**. They in turn will antagonize the anti-resection function of the Shieldin complex, thus stimulating resection processivity **(3)**. Once resection is fully activated and the long-range resection is engaged, *de novo* transcription would create ncRNAs that can pair with the ssDNA, forming DNA:RNA hybrids **(4)**. Such hybrids will not affect resection and will facilitate a full DDR. The presence of such structures might block RPA loading. Thus, elimination of the RNA will be required for activation of homologous recombination and limit DNA end resection **(5)**. In all cases, the polarity of the DNA and RNA is shown. DDR, DNA damage response.

On the other hand, the formation of persistent DNA:RNA hybrids or R-loops at the sites of the break seems to play both positive and negative roles during HR, even earlier to the strand invasion step (Ohle et al., 2016; Cohen et al., 2018; Costantino and Koshland, 2018; Lu et al., 2018; García-Muse and Aguilera, 2019; Jimeno et al., 2019; Paull, 2019; Puget et al., 2019; Domingo-Prim et al., 2020). The role of those R-loops is still controversial and requires further study for clarification. Although this is a very hot topic currently in the field of DNA repair, due to length constraints in this review, we will only summarize some general findings briefly at this point (Figure 2), and later in the context of RNA modifications, that is, the focus of this manuscript (see below). Especially, the role of R-loops in DSB repair and their consequences have been extensively discussed by others and us in recent reviews (for further details, see Jimeno et al., 2019; Puget et al., 2019; Domingo-Prim et al., 2020). For example, in *Schizosaccharomyces pombe*, it has been shown that DNA:RNA hybrids are required for DNA end resection (Ohle et al., 2016) and also that persistent R-loops block the resection prompting asymmetric DNA end processing (Costantino and Koshland, 2018). In human cells, an increase of R-loops close to DSBs located at transcribed regions has also been observed (Cohen et al., 2018; Lu et al., 2018; Bader and Bushell, 2020). We have previously suggested an interpretation of all the published data (Jimeno et al., 2019) that is shown in Figure 2. We propose that the accumulation of the DNA:RNA hybrids has different impacts on the DNA resection process depending on the localization in time and space of such structures. If pre-existent R-loops are present at the break locus before resection starts, they might act as a roadblock and hamper the resection processivity (Figure 2(1)). Then, the activities of proteins such as the helicase SETX would be required in order to facilitate the resection. On the contrary, R-loops close to DSBs can also recruit Rad52 and BRCA1, in turn counteracting the anti-resection activity of the Shieldin complex (Figure 2(2)). This will expedite DNA end processivity (Figure 2(3)). Finally, after the DNA short-range resection has switched to the long-range resection, *de novo* transcription of the ssDNA would create DNA:RNA hybrids that will not interfere with the DNA processing but might be required for a full DDR (Figure 2(4)). However, the removal of the RNA molecule bound to the ssDNA would be required to allow RPA loading onto ssDNA (Figure 2(5)).

## RNA Modifications in DNA Repair

RNA modification is emerging as another important layer of epigenetic regulation. It consists of the posttranscriptional chemical modification of the RNA molecule by different enzymes, creating a plethora of possible alternative epitranscriptomic signatures (Table 1).

**TABLE 1:** Principal RNA modifications and their connection with DNA repair.

**Modification**	**Writers**	**Readers**	**Erasers**	**Connection with DNA repair**
m6A	METTL3- METTL14 METTL16 METTL5 ZCCHC4	YTHDF1 YTHDF2 YTHDF3 YTHDC1 YTHDC2	ALKBH5 FTO	Abakir et al., 2020; Zhang et al., 2020a
m1A	TRMT10 TRM6-TRM61 METTL8	YTHDF2	ALKBH1 ALKBH3	Li et al., 2016; Brickner et al., 2020; Svobodová Kovaříková et al., 2020
m5C	NSUN1 to NSUN7 TRDMT1	ALYREF		
m3C	METTL2 METTL6 METTL8		ALKBH1 ALKBH3	Brickner et al., 2020
m7G	METTL1 METTL8 WBSCR22 RNMT			Brickner et al., 2020
ADP-ribosylation	PARP10 PARP11 PARP15 TRPT1			
A-to-I deamination	ADAR1 ADAR2 ADAT2 ADAT3			Jimeno et al., 2021
ψ	PUS1 to PUS10 PUSL7 RPUSD1 to RPUSD4 DKC1			
mcm5U	ELP1 ELP3 ALKBH8 CTU1 CTU2			Fu et al., 2010; Zdzalik et al., 2014

Ribosomal RNA (rRNA) and transfer RNA (tRNA) are heavily modified RNA molecules (Roundtree et al., 2017), but in recent years, modifications in other RNA families, such as mRNA, micro RNA (miRNA), or lncRNA, have been readily observed (Shelton et al., 2016; Zhao et al., 2016; Esteller and Pandolfi, 2017). More than 100 different RNA modifications have been found in RNA, N6-methyladenosine (m6A), N1-methyladenosine (m1A), 5-methylcytosine (m5C), internal 7-methylguanosine (m7G), 2′-O-methylation (2′-OMe), pseudouridine (ψ), uridylation, ADP-ribosylation, and adenosine deamination to inosine being some of the most common ones (Barbieri and Kouzarides, 2020). For some of these modifications, the proteins that catalyze the modifications (*writers*), the ones that remove the modifications (*erasers*), and the ones that bind specifically to these modifications (*readers*) have been identified. The most prominent examples of RNA modifications, their writers, readers, and erasers, and their connection with DNA repair can be found in Table 1.

As an example of how some RNA modifications work, we can take the methylation of adenosine at position 6 to give N6-methyladenosine (m6A), arguably the best studied of them. First, a *writer* complex modifies the RNA substrate. In the case of m6A, it is mostly dependent on the methyltransferase complex formed by METTL3 and METTL14 (Liu et al., 2014) although other writers exist (Pendleton et al., 2017; Ma et al., 2019; van Tran et al., 2019). In agreement with an epitranscriptomic function, m6A methylation is reversible by the action of an *eraser*, in this case, the demethylases FTO (fat mass and obesity-associated protein) and ALKBH5 (Jia et al., 2011; Zheng et al., 2013). On the other hand, in order to be erased or to perform its varied functions, when a RNA presents m6A, it has to be recognized by a *reader*. Five components of the YTH domain family (YTHDF1-3 and YTHDC1-2), insulin-like growth factor 2 mRNA-binding protein (IGF2BP), heterogeneous nuclear ribonucleoproteins A2/B1(HNRNPA2B1), and proline-rich and coiled-coil containing protein 2A (PRRC2A) can bind to RNAs bearing the m6A modification (Alarcón et al., 2015; Huang et al., 2018; Liao et al., 2018; Wu et al., 2019) and elicit different responses that have an impact in general on RNA metabolism and other processes such as the ones discussed under DNA repair.

**FIGURE 3:**
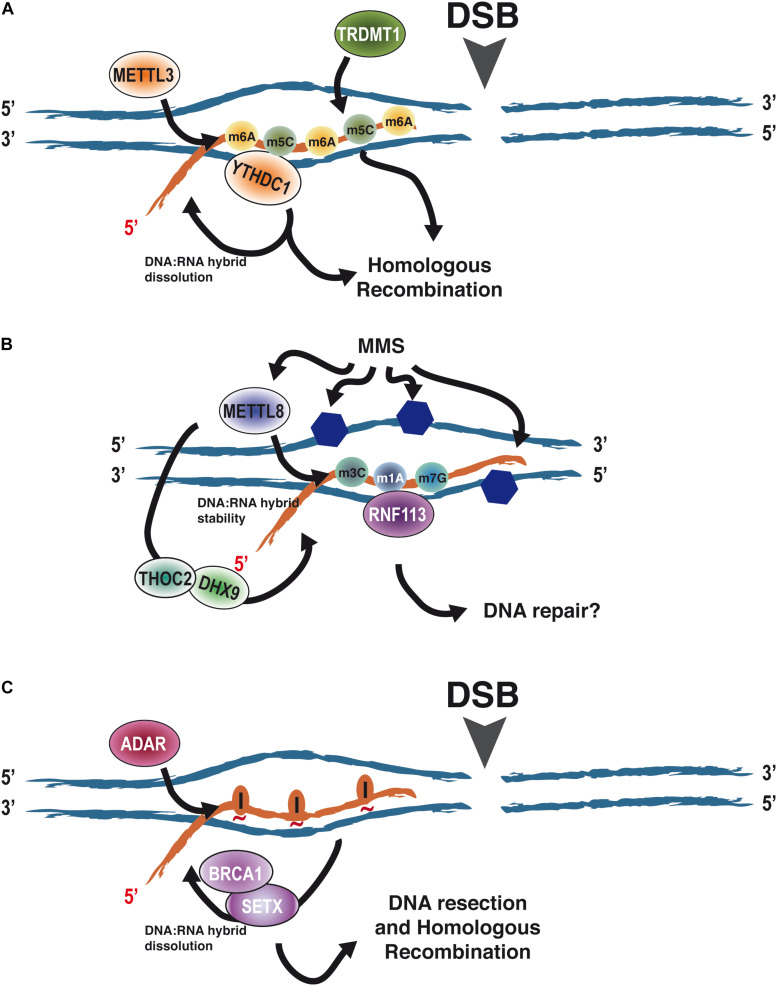
The role of RNA modifications in the context of DNA:RNA hybrids on DNA repair. **(A)** RNA modifications such as m6A and m5C have been shown to accumulate upon the appearance of a DNA DSB on DNA:RNA hybrids due to the recruitment of the writers METTL3 and TRDMT1. Such modifications promote homologous recombination. At least in the case of m6A, this relies on the regulation of DNA:RNA hybrid stability through the eraser YTHDC1. **(B)** MMS causes the alkylation of DNA (blue hexagons) and the induction of the modifications m3C, m1A, and m7G on RNA by METTL8. On the other hand, METTL8 has been proposed to modulate DNA:RNA hybrid stability through THOC2 and DHX9, thus opening the possibility of such modifications happening in the context of R-loops. Furthermore, RNF113 has been shown to interact with m3C, m1A, and m7G after MMS treatment, stimulating DNA repair. Whether, in addition to its role in the spliceosome, this happens in the context of R-loops is an open question. **(C)** The appearance of a DSB stimulates the transient recruitment of ADAR proteins. Its A-to-I editing activity is required to facilitate DNA:RNA hybrid removal by SETX and BRCA1 prior to DNA end resection. We propose that this is mediated by loosening the interaction between the RNA and DNA moieties as inosines will not pair with the opposing thymines (red tilde). Abbreviations: adenosine deaminases acting on RNA.

Recently, it has become clear that RNA modifications also affect DNA repair (Ketley and Gullerova, 2020) (Table 1). The first example was the role of m6A RNA methylation in the repair of DNA damage induced by UV facilitating the rapid recruitment of Pol κ to UV-induced DNA damage (Xiang et al., 2017). Roles of m6A RNA methylation in DSB repair have been also described (Zhang et al., 2020a). Indeed, the RNA methyltransferase METTL3, the m6A RNA reader YTHDC1, and m6A-modified RNA accumulate at DSBs promoting HR (Figure 3A). This effect seems to depend on the modulation of stability of DNA:RNA hybrids at DSBs (Zhang et al., 2020a). Along the same lines, Abakir et al. (2020) in a study non-related with DSB induction, showed that most DNA:RNA hybrids are, indeed, m6A modified and that such RNA modifications on R-loops are important for the maintenance of genetic stability in human cells. However, other studies showed that RNA bearing the m6A modification promotes the formation of co-transcriptional R-loops at transcription termination sites to avoid readthrough by the RNA polymerase II (Yang et al., 2019). Albeit these two studies are at odd in the pro- or anti-stability of m6A for the R-loop, something that should be clarified further, they clearly involve such modification in R-loop metabolism. Furthermore, 5-methylcytosine accumulates at damaged sites and helps DNA repair. As for m6A, the m5C role in this process seems to happen in the context of R-loops and requires transcription and the recruitment of a *writer* factor, TRDMT1, at sites of DNA damage. Importantly, in the absence of TRDMT1, repair by HR is hampered (Chen et al., 2020) (Figure 3A).

Less clear are the connections of other RNA modifications with the repair of the DNA molecule. H_2_O_2_ treatment, which causes DNA oxidation, causes specific changes in the patterns of N1-methyladenosine on RNA (Li et al., 2016). Additionally, UV-irradiation decreases the cellular levels of m1A in RNAs (Svobodová Kovaříková et al., 2020). Strikingly, this global RNA demethylation is carried out by the *eraser* ALKBH3, an enzyme that has been involved in DNA repair (Dango et al., 2011; Mohan et al., 2019). Furthermore, MMS treatment induces the association of RNF113A, a ubiquitin ligase involved in the repair of alkylating agents (Brickner et al., 2017), with RNAs harboring the three major MMS-induced modifications mediated by METTL8 (m3C, m1A, and m7G), suggesting a role of such RNA modifications in response to DNA damage caused by alkylating agents (Brickner et al., 2020). Albeit so far RNF113A role in DNA repair seems to be connected with its role in the spliceosome (Shostak et al., 2020). The fact that METTL8 has been also proposed to modulate the R-loop stability through its association with THOC2 and DHX9 (Zhang et al., 2020b) opens the possibility that m1A and/or other METTL8-mediated RNA modifications might also play a role in DNA repair of different DNA lesions as well as DDR by regulating R-loop biology at the breaks (Figure 3B).

Human ALKBH8, which catalyzes methylation of tRNA to form mcm5 U, has also been connected with the DDR. Not only its expression is upregulated upon exposure to DNA damaging agents in an ATM0 dependent fashion but also its depletion sensitizes cells to MMS and bleomycin treatments (Fu et al., 2010). Interestingly, such a role is broadly conserved in evolution (Zdzalik et al., 2014). This function has been associated so far with tRNA modification but opens the possibility that the effect could also be mediated by altering other RNAs.

Additionally, it has been recently demonstrated that RNA can be a target of reversible mono-ADP-ribosylation. Indeed, PARP10, PARP11, and PARP15, as well as a PARP homolog TRPT1, are able to ADP-ribosylate RNA-phosphorylated ends. This ADP-ribosylation of RNA is efficiently reversed *in vitro* by several ADP-ribosylhydrolases (Munnur et al., 2019). PARylation of proteins by PARP1, 2, and 3 has important roles in DNA repair, both in single-strand breaks (SSBs) and DSBs repair (Caldecott, 2014). So, the role of ADP-ribosylated RNA in the response to DNA breaks is worth exploring in the future.

Another form of RNA modification is RNA editing, caused by the deamination of either C or A, to change the RNA sequence to either U or I, respectively. Unlike other RNA modifications, these are not reversible. However, on the other hand, it can elicit many different responses, such as changes in RNA stability, splicing, or even altering the coding sequence. In principle, every mammalian transcript can be subjected to RNA editing (Gallo et al., 2017). Interestingly, we have observed that A-to-I deamination by ADAR proteins is required for efficient DNA end resection and HR (Jimeno et al., 2021). As for m6A and m5C, it seems to happen in the context of DNA:RNA hybrids, as the phenotype is suppressed by RNaseH overexpression, and is essential for R-loop dissolution and clearance prior DNA end resection. Indeed, our data suggest DNA:RNA hybrids, resulting either from pre-existing transcription or from newly transcribed RNA after DNA damage, block DNA resection. Then, transient recruitment of ADAR to sites of DNA DSBs where its adenine deamination activity is required to, in turn, facilitate hybrid removal by SETX and BRCA1 in order to expedite resection (Jimeno et al., 2021) (Figure 3C).

### The Role of RNA Modifications on DSB Repair by DNA:RNA Hybrids Stability

When RNA acts as a donor of information to fulfill HR, a DNA:RNA hybrid has to be formed. These DNA–RNA structures also form as a consequence of damage-induced transcription at DSBs sites. Thus, the formation, stability, and regulation of these DSBs-related R-loops play a central role in many aspects of the DNA repair process (Ohle et al., 2016; Costantino and Koshland, 2018; García-Muse and Aguilera, 2019; Paull, 2019; Puget et al., 2019). As mentioned before, the impact of DNA:RNA hybrids on DNA resection is still under debate since not only negative but also positive roles have been proposed for those structures on this first step of the HR process (Ohle et al., 2016; Costantino and Koshland, 2018; Jimeno et al., 2019; Domingo-Prim et al., 2020).

One way to reconcile these observations is that R-loops might vary their impact on DNA end resection and HR, depending on modifications on the RNA moiety. Thus, RNA modifications of R-loops will bring an extra epitranscriptomic layer of control to DSB repair, affecting the balance between resection-mediated repair, such as HR, and those repair pathways that are resection-independent like NHEJ. This new level of regulation might rely on the stabilization/destabilization of R-loops at DSBs as a response to different cellular cues, such as DNA chromatin structure, cell cycle position, time after the break formation, etc. In this model, R-loops will initially block DNA end resection, providing an initial window of opportunity for NHEJ to act, but if the break cannot be repaired or in the presence of pro-HR signals, the modification of the RNA will facilitate its removal, allowing resection to proceed. Moreover, prior to DNA:RNA dissolution, such modifications could enhance the recruitment of HR proteins, thus explaining why some authors have seen a positive effect of R-loop formation in recombination. So, modifications such as the aforementioned m6A, m5C, or A-to-I editing will facilitate HR (Chen et al., 2020; Zhang et al., 2020a; Jimeno et al., 2021) (Figure 3). To add an extra layer of complexity, it is known that these RNA modifications crosstalk, and, indeed, they regulate each other reciprocally (Xiang et al., 2018). In this scenario, an “R-loop code” based on several RNA modifications could fine-tune DSB repair, controlling the licensing of DNA end resection, thus channeling the DNA ends toward the most appropriate repair route.

### RNA Modification, DNA Repair, and Cancer

Defective DNA repair is recognized as a hallmark of cancer cells, as it will increase the mutation rate associated with tumorigenesis (Hanahan and Weinberg, 2011). Strikingly, RNA modifications have also been associated with cancer development (Barbieri and Kouzarides, 2020). Despite that several roles of RNA modification have been already linked with cancer development, we wonder if part of this connection could be due to defective DNA repair when RNA modification levels are altered.

For example, the m6A *writer* METTL3 has been found to be overexpressed in several cancer types (Lin et al., 2016; Barbieri et al., 2017; Vu et al., 2017; Chen et al., 2018). METTL3-mediated m6A methylation of mRNAs promotes the expression of oncogenes, and the degradation of the mRNAs coding for tumor suppressors participates in essential processes such as cell proliferation, maintenance of undifferentiated phenotype, or the epithelial to mesenchymal transition (Lin et al., 2016; Barbieri et al., 2017; Chen and Yu, 2018; Barbieri and Kouzarides, 2020). But as mentioned, METTL3 also plays a relevant role in DSB repair, thus opening the possibility that some of those phenotypes are indirect, acquired by mutations due to incorrect DNA repair.

The ADAR-mediated adenosine-to-inosine editing has also been linked to several types of cancers. High levels of ADAR1 suppress the immune response and promote cell growth in cancer cells. Therefore, targeting ADAR1 has been proposed as a potential strategy to prevent resistance to immunotherapy (Ishizuka et al., 2019). On the contrary, ADAR2 has been reported to have mainly a tumor suppressor role in cancer. ADAR2 levels are downregulated in the glioblastoma and the high-grade astrocytomas (Cenci et al., 2008; Tomaselli et al., 2013; Gallo et al., 2017). As ADAR-deficient cells are defective in DNA repair (Jimeno et al., 2021), it will be worth exploring if those tumors with overexpression or downregulation of these factors might benefit from specific DNA damage–inducing cancer treatments.

## Author Contributions

SJ with the help of FB browsed the literature and collected the source data. SJ with PH wrote the manuscript. All authors contributed to the article and approved the submitted version.

## Conflict of Interest

The authors declare that the research was conducted in the absence of any commercial or financial relationships that could be construed as a potential conflict of interest.
